# Clinical efficacy and safety of percutaneous drainage for post-operative fluid collection in patients with bladder cancer undergoing radical cystectomy and urinary diversion

**DOI:** 10.1097/MD.0000000000036488

**Published:** 2023-12-08

**Authors:** Chang Hoon Oh

**Affiliations:** a Department of Radiology, Ewha Womans University Mokdong Hospital, College of Medicine, Ewha Womans University, Seoul, Republic of Korea.

**Keywords:** abscess, bladder cancer, cystectomy, percutaneous drainage

## Abstract

To evaluate the success rates of percutaneous drainage for fluid collection after radical cystectomy, with emphasis on factors affecting the clinical success, including lesion, patient, and procedure characteristics. In this retrospective study, 31 percutaneous drainage catheters were placed in 29 consecutive patients between January 2021 and September 2023. Most fluid collections formed near the uretero-ileal anastomosis site in the right pelvic cavity (80.6%). The technical success rate was 100%. The primary and final clinical success was 80.6% and 96.9%, respectively. Lymphoceles notably increased the primary clinical failure risk (odds ratio and 95% confidential interval: 22.667 (1.839–279.366), *P* = .015). Significant differences were observed between transabdominal and transgluteal approaches in terms of fluoroscopic time, dose, and location. Leakage indications on computed tomography prompted differing interventions, but all achieved final clinical success. Percutaneous drainage for post-operative fluid collection is safe and effective in patients with radical cystectomy and urinary diversion.

## 1. Introduction

Bladder cancer is one of the most common malignancies and a significant cause of cancer-related deaths worldwide.^[1]^ Approximately one-fourth of patients with bladder cancer present with muscle-invasive bladder cancer requiring multidisciplinary oncologic treatment.^[2]^ Radical cystectomy with pelvic lymph node dissection provides the best cancer-specific survival for bladder cancer, with 10-year recurrence-free survival rates of 50% to 59% and overall survival rates of approximately 45%.^[3,4]^

Currently, the following major techniques are used for urinary diversion after radical cystectomy: incontinent cutaneous diversion (conduit) and continent diversion to the intact native urethra (orthotopic neobladder).^[5]^ Early complications, defined as occurring either during hospitalization or within the first 30 days after surgery, occur in 20% to 57% of patients.^[6]^ Early complications include urine leakage, ureteral obstruction, post-operative fluid collection, and fistulas between ileal conduits and nearby organs.^[5]^

Fluid collection is frequently seen in the early post-operative period after radical cystectomy. Among these, urinomas and abscesses usually require prompt percutaneous drainage unless they are small. Seromas, hematomas, and lymphoceles require percutaneous drainage when they become symptomatic, or infected, or when they obstruct nearby structures such as the ureter, bowel, or ileal conduit.^[5]^

Percutaneous drainage can obviate open surgery in patients with comorbidities or stabilize the patient condition before open surgical reintervention. Technical approaches for pelvic fluid or abscess drainage and clinical success rates on patient series were widely reported in the literature; however, studies on factors affecting clinical success are limited.^[7]^ The purpose of this study was to evaluate the success rates of percutaneous drainage for fluid collection after radical cystectomy, with emphasis on factors affecting clinical success, including lesion, patient, and procedure characteristics.

## 2. Materials and methods

### 2.1. Patient population

**Figure 1.**
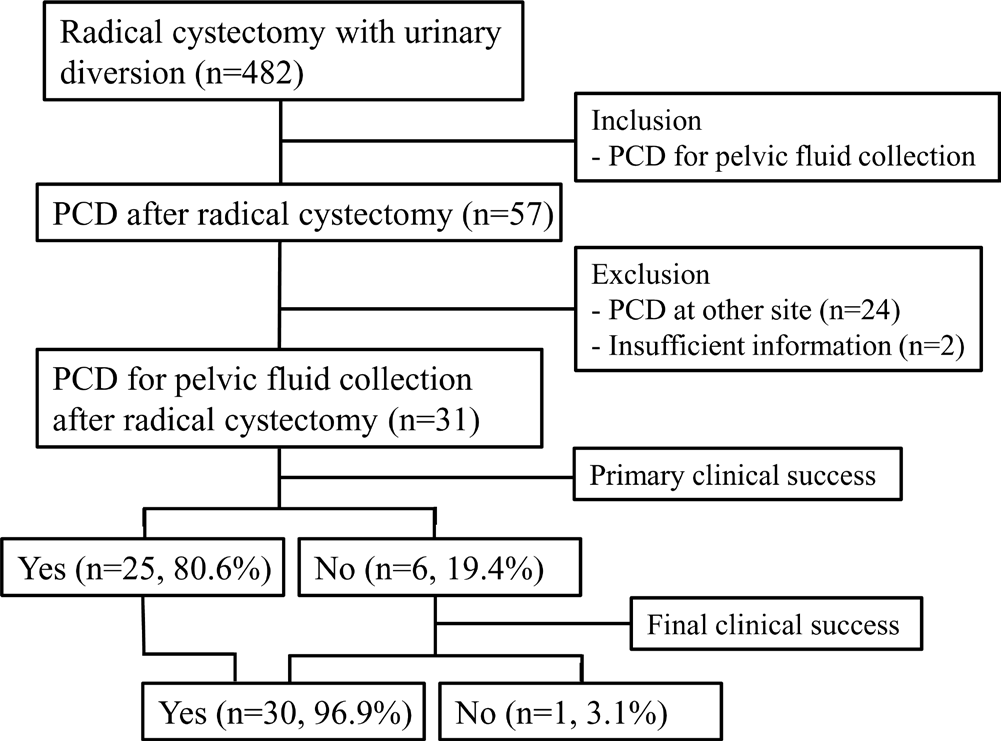
Flowchart of percutaneous drainage catheter placement in patients with radical cystectomy and urinary diversion. PCD = percutaneous catheter drainage.

This retrospective, single-center study was approved by the institutional review board, which waived the need for obtaining informed consent from the patients (Approval no. EUMC 2023-10-014). All patients who underwent radical cystectomy owing to bladder cancer with an ileal conduit or an ileal orthotopic neobladder for bladder cancer were included. Patients who had undergone percutaneous catheter drainage (PCD) at our hospital between January 2021 and September 2023 were enrolled in the study. A total of 57 cases of PCD were performed after radical cystectomy and 26 cases were excluded owing to procedures on areas other than the pelvis or insufficient information. A total of 31 PCD were performed under ultrasound or cone-beam computed tomography (CT) guidance in 29 consecutive patients. The inclusion criteria were patients aged 40 to 90 years receiving PCD owing to post-operative fluid collection in the pelvic area after radical cystectomy with an ileal conduit or an ileal orthotopic neobladder. The exclusion criteria were life expectancy of less than 3 months, drainage catheter inserted in areas not associated with radical cystectomy, and poor general health status (Eastern Cooperative Oncology Group performance status grade 4) (Fig. 1).

### 2.2. Procedure

In all cases, percutaneous abscess drainage was performed by interventional radiologist staff with 3, 10, and 22 years of experience. International normalized ratio above 1.5 was corrected with oral or intravenous vitamin K administration or with fresh frozen plasma infusion, and platelet counts <50.000/mcL required prophylactic platelet transfusion. All catheters were placed with the Seldinger technique via transabdominal or transgluteal routes according to location and adjacent structures of the fluid collection.

In the transabdominal approach, a local anesthetic (up to 10 mL of lidocaine) was injected at the skin site and along the tract. The fluid was collected using a 21-gauge Chiba needle under ultrasound guidance. Once punctured, a 0.016-inch floppy-tipped guidewire was advanced through the lumen of the needle, and the needle was then withdrawn, leaving the distal tip of the wire coiled in the collection. Subsequently, 5-F yellow sheath and 0.035-inch guidewire were inserted. Fascial dilators were then advanced over the wire with a stepwise increase in diameter to dilate the intended track of the catheter. The depth of the cavity was marked on the side of the dilators to avoid excessive advancement and guide wire dislocation. Kinking the guide wire while using the stiff dilators was strictly avoided. Once the track was dilated, the drainage catheter (8.5–12 French, pigtail catheter) assembled with stiffener was advanced along the wire to reach the collection. After ensuring that the catheter was positioned correctly into the collection, the stiffener was removed with the guiding wire and the catheter was fixed to the skin.

The transgluteal approach was performed with the patient in the prone position. After the acquisition of the initial cone-beam CT for assessing the depth of and approach to the pelvic abscess, a 21-gauge needle was advanced into the fluid collection. The position of the needle in the cavity was confirmed with cone-beam CT. The subsequent procedure was carried out in the same manner as the transabdominal approach with the Seldinger technique.

### 2.3. Follow-up

The follow-up imaging was performed when the amount of drained fluid through the drainage catheter was minimal. The drainage catheter was removed when the CT showed that the volume of the existing cavity had significantly reduced or the patient clinical and laboratory responses were good. However, if the fluid collection cavity size did not decrease significantly or the patient was not improving clinically, drainage catheter exchange and repositioning were performed.

### 2.4. Study endpoints

Primary clinical success was defined as the resolution of fluid collection without any additional procedures through the initially inserted PCD. Final clinical success was defined as complete resolution of fluid collection and no further intervention regardless of the number of procedures conducted. Technical success was defined as successful drainage catheter placement at the desired location. Complications were classified as minor or major according to the Society of Interventional Radiology guidelines.^[8]^

### 2.5. Statistical analysis

The statistically analyzed variables and their details were as follows: patient characteristics: age, gender, type of urinary diversion, time to operation (between PCD and radical cystectomy with urinary diversion), duration of catheter drainage, and hospital stay; fluid collection characteristics: CT finding and structure (complex multilocular or simple unilocular), density in CT (housefield unit), location, drainage fluid property (urinoma, seroma, lymphocele, and hematoma), and presence or suspicion of leakage; procedure characteristics: guidance method (ultrasound or CT) and access route (transabdominal or transgluteal). Continuous variables are presented as the mean ± standard deviation and were compared using the Student *t* test. Categorical variables were compared using the chi-squared test or Fisher exact test. Univariate logistic regression analysis of the risk factors for the primary clinical failure was performed. All statistical analyses were performed using SPSS software version 20.0, with *P* values <.05 indicating statistical significance.

## 3. Results

Baseline demographics and clinical data of the study patients are shown in Table 1. A total of 31 percutaneous drainage catheter placements were included. There were 21 male and 10 female cases with an average age of 63.3 ± 10.3 years. The interval from radical cystectomy to PCD implementation was 29.1 ± 30.9 days. Twenty-nine cases were orthotopic neobladders and 2 were ileal conduits. On CT, 14 were simple fluid collections (45.2%) and 17 were complicated fluid collections (54.8%). After catheter insertion, 15 aspirations were clear (48.4%), 10 were turbid (32.3%), and 6 had pus (19.3%). Regarding fluid structure, 12 appeared simple-unilocular (38.7%) and 19 complex-bilocular (61.3%), with the complex-bilocular appearance being more common. The average fluid density was 13.2 ± 5.7 housefield unit. Most abscesses or fluid collections formed near the uretero-ileal anastomosis site (peri-anastomosis area) in the right pelvic cavity (25 cases, 80.6%), with 4 cases in the left pelvic cavity (12.9%) and 2 cases located in the presacral or perirectal area (6.5%). Depending on the lesion location and surrounding structures such as bowels and large vessels, a transabdominal or transgluteal approach was chosen. Twenty-four cases underwent the transabdominal (77.4%) and 7 cases the transgluteal approach (22.6%). Of the 7 cases with a transgluteal approach, 2 were in the presacral or perirectal area, 2 in the left pelvic cavity, and 3 near the uretero-ileal anastomosis site in the right pelvic cavity. The average catheter drainage duration was 11.3 ± 10.6 days, and the hospital stay duration was 25.6 ± 16.7 days.

**Table 1:** Baseline demographics and clinical data of the study patients and assessment of primary clinical success.

Characteristics	N (%)	Primary clinical success		
		Yes (n = 25)	No (n = 6)	*P*
Sex				.301
Male	21 (67.7)	18	3	
Female	10 (32.3)	7	3	
Age, yr (range)	63.3 ± 10.3	62.8 ± 10.6	65.5 ± 9.4	.566
Time to operation (d)	29.1 ± 30.9	23.7 ± 22.8	51.7 ± 49.8	.233
Urinary diversion				.257
Neobladder	29 (93.5)	24	5	
Ileal conduit	2 (6.5)	1	1	
Computed tomography finding				.517
Simple fluid	14 (45.2)	12	2	
Complicated fluid collection	17 (54.8)	13	4	
Aspiration				.220
Clear	15 (48.4)	14	1	
Turbid	10 (32.3)	7	3	
Pus	6 (19.3)	4	2	
Fluid cavity structure				.763
Simple-unilocular	12 (38.7)	10	2	
Complex-bilocular	19 (61.3)	15	4	
Presence or suspicion for leakage				.853
Yes	6 (19.4)	5	1	
No	25 (80.6)	20	5	
Drainage fluid property				.013
Urinoma	6 (19.3)	5	1	
Seroma	18 (58.1)	17	1	
Lymphocele	7 (22.6)	3	4	
Hematoma	-	-	-	
Fluid density (housefield unit, HU)	13.2 ± 5.7	13.1 ± 5.5	13.7 ± 7.0	.817
Location				.753
Perianastomosis area, Right	25 (80.6)	20	5	
Left pelvic cavity	4 (12.9)	3	1	
Presacral/perirectal area	2 (6.5)	2	0	
Access route				.483
Transabdominal	24 (77.4)	20	4	
Transgluteal	7 (22.6)	5	2	
Duration of catheter drainage (d)	11.3 ± 10.6	11.4 ± 10.2	10.3 ± 8.1	.839
Hospital stay (d)	25.6 ± 16.7	23.5 ± 11.8	38.0 ± 34.9	.470
Technical success	32 (100)	-	-	-
Primary clinical success	25 (80.6)	-	-	-
Final clinical success	30 (96.8)	-	-	-

The technical success rate was 100% without any complications during the procedure. Among them, 25 cases (80.6%) showed primary clinical success; excluding 1 case, 30 cases (96.9%) achieved final clinical success. Primary clinical success was found to be significant only in relation to drainage fluid properties (*P* = .013). Logistic regression analysis revealed that the presence of lymphoceles significantly increased the risk of primary clinical failure (odds ratio and 95% confidence interval: 22.667 (1.839–279.366), *P* = .015). Among the 7 cases of lymphocele, primary clinical failure was observed in 4 cases. However, in 3 of these cases, final clinical success was achieved following drainage catheter exchange or removal followed by re-insertion. However, in 1 case, even after performing intranodal lymphangiography using lipiodol suspecting lymphatic leakage, it showed a negative finding and sequential embolization was not performed. Despite undergoing catheter exchange with upsizing (8.5-F →10.2-F), the drainage volume did not decrease, and the size and extent did not reduce on follow-up CT, resulting in final clinical failure (Table 2).

**Table 2:** Primary clinical failure patients after percutaneous drainage catheter placement undergone radical cystectomy with urinary diversion.

Age/Sex	Operation interval (d)	Location	Structure	Access route	Presence or suspicion for leakage	Reason for primary failure	Final clinical success	Additional intervention
M/60	26	Perianastomosis	Simple-unilocular	Transabdominal	X	Poor drainage	O	Successful drainage after catheter exchange within 7 d
M/77	88	Perianastomosis	Complex-bilocular	Transabdominal	X	Recurrent	O	Re-insertion of drainage catheter and successful drainage within 21 d
M/72	6	Perianastomosis	Complex-bilocular	Transabdominal	X	Poor drainage	O	Successful drainage after catheter exchange with upsizing (8.5-F → 10.2-F) within 5 d
F/70	134	Left pelvic cavity	Complex-bilocular	Transgluteal	X	Persistent drainage	X	Persistent drainage after catheter exchange (8 and 44 d after fist insertion). No definite extravasation on intranodal lipiodol lymphangiography (44 d after first insertion)
F/51	13	Perianastomosis	Simple-unilocular	Transabdominal	X	Recurrent	O	Re-insertion of drainage catheter and successful drainage within 7 d
F/63	43	Perianastomosis	Complex-bilocular	Transgluteal	O	Poor drainage	O	Successful drainage after catheter change within 10 d

Six patients had CT results indicating the presence or suspicion of leakage. Among them, 3 patients had wall defects accompanied by contrast media leakage; all underwent percutaneous nephrostomy (PCN). In the other 3 cases, without any pronounced wall defect and only with minimal leakage, only a drainage catheter was inserted without PCN placement, and all showed final clinical success. Furthermore, when comparing the transabdominal and transgluteal approaches, significant differences were found in fluoroscopic time (1.17 ± 0.69 vs 3.31 ± 1.71 minutes, *P* = .015), dose (229.7 ± 296.8 vs 4104.3 ± 1806.2 µGym^2^, *P* = .001), and location (peri-anastomosis area, right pelvic cavity: 22 vs 3; left pelvic cavity: 2 vs 2; presacral or perirectal area: 0 vs 2, *P* = .005) (Table 3).

**Table 3:** According to approach route of percutaneous drainage catheter in patients undergone radical cystectomy with urinary diversion.

	Approach route		
	Transabdominal (n = 24)	Transgluteal (n = 7)	*P*
Fluoroscopic time (min)	1.17 ± 0.69	3.31 ± 1.71	.015
Dose (uGym^2^)	229.7 ± 296.8	4104.3 ± 1806.2	.001
Computed tomography finding (%)			.889
Simple fluid	11 (45.8)	3 (42.8)	
Complicated fluid collection	13 (54.2)	4 (57.2)	
Fluid cavity structure (%)			.531
Simple-unilocular	10 (41.7)	2 (28.6)	
Complex-bilocular	14 (58.3)	5 (71.4)	
Fluid density (housefield unit, HU)	13.3 ± 5.1	13.0 ± 7.5	.882
Location (%)			.005
Perianastomosis area, right	22 (91.7)	3 (42.8)	
Left pelvic cavity	2 (8.3)	2 (28.6)	
Presacral or perirectal area		2 (28.6)	
Duration of catheter drainage (d)	11.7 ± 11.3	9.2 ± 6.7	.638
Hospital stay (d)	25.0 ± 17.9	24.8 ± 11.2	.977
Primary clinical success (%)	20 (83.3)	5 (71.4)	.483
Final clinical success (%)	24 (100)	6 (85.7)	.060

## 4. Discussion

According to the results of this study, percutaneous drainage for post-operative fluid collection had a high clinical success rate (primary clinical success: 81.3%, final clinical success: 96.9%) and no major complications. The only factor affecting primary clinical success was the drainage fluid properties. Lymphoceles were observed in 7 cases, and 3 of these (42.9%) had primary clinical success, whereas the other post-operative fluid collections showed 91.6% primary clinical success (*P* = .013). Treatment options for pelvic lymphocele can vary depending on the severity of the clinical symptoms, the treating urologist, and patient factors such as location, lymphocele size, whether the lymphocele is infected, the overall health condition of the patient, and lymphocele recurrence.^[9–11]^ According to a recent meta-analysis, the success rate (95% CI) for post-operative lymphocele after PCD is 0.612 (0.490–0.722). Sclerotherapy with percutaneous catheter drainage is recommended in the treatment of post-operative pelvic lymphoceles because the success rate (95% CI) of PCD with delayed sclerotherapy is 0.890 (0.781 − 0.948).^[12]^ Because of its lower complication and recurrence rates, shorter treatment duration, and fewer reinterventions, embolization is the treatment of choice. In our study, we defined primary clinical success as a complete resolution of fluid collection with the first drainage catheter insertion without drainage catheter exchange. This might have made the success rate seem lower, but we observed a 6/7 (85.7%) final clinical success rate. This result is either on par with or better than previous studies. Perhaps due to the high viscosity of lymphocele, the drainage catheter exchange may be effective, which could explain the relatively high final clinical success despite low primary clinical success rate. However, large cohort studies are necessary.

In this study, 1 case had no definite extravasation of lipiodol in the intranodal lymphangiography, which ultimately resulted in clinical failure despite drainage catheter exchange after the procedure. Occlusion of the lymphatic inflow within the pelvic lymphocele is crucial for preventing lymphocele recurrence.^[13]^ Chu et al showed that 66.7% (6/9) of cases showed rapid resolution with a median resolution time of only 7 days after a single session of lipiodol lymphangiography, and the remaining patients improved after the second session.^[14]^ Another study also showed a 50% success (7/14) with a single session of lipiodol lymphangiography, and 75% (3/4) of the remaining patients showed clinical success after the second session.^[15]^ The patient with clinical failure in this study was subsequently lost to follow-up and could not proceed with the second session of lipiodol lymphangiography. However, based on previous studies, it is speculated that there might have been some clinical efficacy. Although there have been many studies on intranodal lymphangiography using lipiodol, large cohort studies are necessary.

Following radical cystectomy, orthotopic neobladder creation for urinary diversion is currently well established. Similar to other diversion techniques, this procedure requires a bowel segment; however, it avoids an abdominal stoma and therefore offers an improved quality of life for patients undergoing radical cystectomy.^[16]^ In our institution, orthotopic neobladders are more commonly performed after radical cystectomy than ileal conduits. In our set of cases, the majority (29 cases) received neobladders compared to only 2 cases with ileal conduit. However, the incidence of urinary leak in orthotopic neobladder formation is theoretically higher owing to a longer suture line compared to an ileal conduit.^[17]^ In this study, in the 3 cases where urine leakage was evident, both PCD and PCN were used, and in the 3 cases with only minimal leakage, PCD alone showed complete final clinical success. Although further studies with a larger cohort are needed, treatment using PCD for urinoma proved to be effective.

Although the incidence of post-operative fluid collections caused by urinary leakage, urinomas, seromas, lymphoceles, or hematomas is poorly known, urinary leakage occurs in approximately 4% of patients after urinary diversion.^[18]^ Currently, in our institution, the Urology Department has performed more than 150 to 180 radical cystectomies annually since 2021. Over the 33 months included in our study, 482 radical cystectomy were performed, and among them, only 29 patients (31 cases) required drainage of post-operative fluid collection in the pelvic cavity on follow-up CT, making it a very small number of cases. The volume-outcome relationship for radical cystectomy is clear, with high concordance between high-volume centers showing reduced mortality by as much as 37% after 30 days.^[19]^ Reduced mortality within high-volume centers is related to a reduction in “failure-to-rescue” events following complex surgery and suggests that the perioperative management of patients undergoing radical cystectomy is critical in improving mortality and morbidity in this group.^[20]^

Although the transabdominal approach is the simplest, it may not always be feasible owing to the interposed intestine and other pelvic viscera.^[21]^ For percutaneous drainage of fluid collections that are not accessible with a transabdominal approach, the choice is usually among the transvaginal, transrectal, and transgluteal approaches in women and between the transgluteal and transrectal approaches in men. The choice likely will be based on personal experience with the various approaches and on the relative availability of CT.^[21,22]^ At our institution, in patients in whom a transgluteal approach is possible, it is usually preferred over the transrectal and transvaginal approaches. In this study, the transgluteal approach showed significantly higher values in terms of fluoroscopic time (*P* = .015), dose (*P* = .001), and location (*P* = .005) compared with the transabdominal approach and no major complication. Often, during the transgluteal approach, it is challenging to obtain a clear view with ultrasound, which is why PCD was performed using cone-beam CT guidance, likely resulting in increased fluoroscopic time and dose. However, even in this study, the transgluteal approach to PCD proved to be effective in cases of post-operative fluid collection after radical cystectomy without major complication such as bleeding through cone-beam CT guidance.^[21,23]^

This study has some limitations, mostly resulting from the small number of patients and its retrospective nature. Studies with larger patient cohorts and longer follow-up data are required to verify the safety and efficacy of PCD for post-operative fluid collection after radical cystectomy with urinary diversion. However, our results suggest that percutaneous drainage may have an important role in the treatment of post-operative fluid collection after radical cystectomy by conservative methods.

In conclusion, percutaneous drainage for post-operative fluid collection was safe and effective in patients with radical cystectomy and urinary diversion. However, if the post-operative fluid collection is a lymphocele, a drainage catheter exchange or other interventional treatments such as sclerotherapy or lymphangiography may be required for final clinical success. When using the transgluteal approach, implementation with cone-beam CT can be performed safely and effectively.

## Author contributions

**Conceptualization:** Chang Hoon Oh.

**Data curation:** Chang Hoon Oh.

**Formal analysis:** Chang Hoon Oh.

**Investigation:** Chang Hoon Oh.

**Writing – original draft:** Chang Hoon Oh.

**Writing – review & editing:** Chang Hoon Oh.
